# Angiotensin II Modulates Podocyte Glucose Transport

**DOI:** 10.3389/fendo.2018.00418

**Published:** 2018-07-24

**Authors:** Barbara Lewko, Anna Maryn, Elzbieta Latawiec, Agnieszka Daca, Apolonia Rybczynska

**Affiliations:** ^1^Department of Pathophysiology Faculty of Pharmacy, Medical University of Gdansk, Gdańsk, Poland; ^2^Department of Pathology and Experimental Rheumatology, Faculty of Medicine, Medical University of Gdansk, Gdańsk, Poland

**Keywords:** podocytes, angiotensin II, insulin, glucose transport, GLUT transporters, high glucose

## Abstract

Podocytes play a central role in the maintenance of the glomerular filtration barrier and are cellular targets of angiotensin II (AngII). Non-hemodynamic pathways of AngII signaling regulate cellular function and mediate podocyte abnormalities that are associated with various glomerulopathies, including diabetic kidney disease. In this study we investigated the capacity of AngII to modulate glucose uptake in mouse podocytes expressing the human AT1 receptor (AT1R+) after 5 days of exposure to normal (NG, 5.6 mmol/L) or to high (HG, 30 mmol/L) glucose. Short (30 min) as well as long-term (24 h) incubations with AngII markedly enhanced glucose transport in both NG and HG cells. In podocytes cultured under NG conditions, AngII inhibited insulin-stimulated glucose uptake. Regardless of the presence or absence of AngII, no effect of insulin on glucose uptake was observed in HG cells. Stimulation of glucose transport by AngII was mediated by protein kinase C and by phosphoinositide 3*-*kinase. Glucose dependent surface expression of the glucose transporters GLUT1, GLUT2, and GLUT4 was modulated by AngII in a time and glucose concentration dependent manner. Furthermore, despite its inhibitory effect on insulin's action, AngII elevated the number of podocyte insulin receptors in both NG and HG cultured cells. These findings demonstrate that AngII modulates podocyte basal, as well as insulin-dependent glucose uptake by regulating glucose transporters and insulin signaling.

## Introduction

Angiotensin II (AngII), the major effector hormone of the renin-angiotensin system (RAS), plays a crucial role in the normal physiological maintenance of renal homeostasis. AngII paracrine and endocrine activities are implicated in the development of kidney diseases (1). It is now well established that independent of hemodynamic effects, AngII acts directly on renal cells (2, 3).

However, the reported effects of AngII on glucose uptake in cultured cells are inconsistent. Some studies have shown that basal glucose transport is enhanced by AngII (4–6), suppressed (7–9), or without effect (10). Further, the effects of AngII on insulin-stimulated glucose uptake are dependent on cell type (11, 12).

Podocytes are terminally differentiated cells of an epithelial origin that cover the outer aspect of glomerular capillaries. Their major functions are structural support of capillary loops, preservation of the glomerular filtration barrier, and interaction with other glomerular cells (13). Podocyte injury or loss is associated with the majority of glomerular abnormalities in that these cells are central to the maintenance of a healthy glomerulus (14, 15). Podocytes express AT1 and AT2 receptors, and apart from being exposed to circulating AngII during filtration, they have their own functional intrinsic RAS (16). AngII regulatory as well as deleterious podocyte effects have been demonstrated both *in vitro* and *in vivo* (16–19). Podocyte damage and loss is a recognized feature of diabetic glomerulopathy (20) due to high ambient glucose, which activates the local podocyte RAS resulting in cell damage (21). Glucose is a major energy substrate for podocytes (22). AngII signaling pathways affect podocyte structure and function by multiple mechanisms (23) which may include modifying energy metabolism through glucose dysregulation. In this study, podocytes were exposed to normal and to high levels of glucose and the effect of AngII on basal and insulin-dependent glucose uptake was examined.

Only a fraction of cultured podocytes respond to AngII stimulation. Hence, a previously characterized mouse podocyte cell line was transfected with the human AT1 receptor (AT1R+) as an experimental model for examination of AngII signaling mechanism(s) (24).

## Materials and methods

### Cell culture

Conditionally immortalized mouse podocytes, stably expressing functional human AngII type 1 receptor (AT1R+), and control cells transfected with an empty vector (AT1R-) were a generous gift of Dr. Hsiang-Hao Hsu, Department of Medicine D, University Hospital Muenster, Germany (24). The cells were cultured in RPMI 1640 at 33°C, in the presence of 10% fetal bovine serum (FBS), 100 U/ml penicillin, 0.1 mg/ml streptomycin, and 10 U/ml mouse recombinant γ-interferon (Sigma-Aldrich, Poland). Differentiation was induced by shifting the cells to 37°C. Cell culture was continued for an additional 10–14 days without γ-interferon and with the concentration of FBS reduced to 5%. To maintain stable cell transfection, all culture media contained 75 μg/ml geneticin (G418, Sigma-Aldrich, Poland). All experiments were performed with differentiated podocytes that were cultured for 5 days after initiation by switching the cells to experimental media containing D-glucose at 5.6 mmol/L (normal glucose, NG) or 30 mmol/L (high glucose, HG). The media were prepared using RPMI 1640 without glucose (Sigma-Aldrich, Poland) with osmolarity adjusted by the addition of mannitol. NG and HG media were supplemented with 5% FBS, G418, and antibiotics, as indicated above. Cell viability was assessed by measuring lactate dehydrogenase (LDH) levels as described previously (25), which was not <87%.

### Treatment of podocytes and measurement of glucose uptake

Following serum starvation of cells for 24 h, glucose uptake was measured for 15 min at 37°C in 24-well plates (in triplicate samples), in a total volume of 500 μl of NG or HG media containing 0.5% (v/v) FBS. To determine the initial rate of glucose transport, the cells were pre-incubated for 3 h in glucose-free RPMI 1640 in the absence of FBS. Steady-state glucose transport was measured in cells maintained in serum-free NG or HG. One micro mole per liter AngII was added at 24 h, 3 h, or 15 min before measuring glucose uptake. Three hundred nano mole per liter insulin was added 5 min prior to assay start. To protect AngII from cleavage, incubations were conducted in the presence of the aminopeptidase A inhibitor, amastatin (Sigma-Aldrich, Poland) at a concentration of 10 μmol/L (26, 27). Thirty min before the start of measurement, media was replaced by reaction media (RM) containing RPMI 1640 supplemented with 0.5, 1.0, 5.6, or 30 mmol/L glucose. The measurement of glucose uptake was initiated by adding 1 μCi/ml [^3^H] 2-deoxy-D-glucose (^3^H-2DG, 29.8 Ci/mmol, NEN-DuPont, Boston, MA, USA). The reaction was stopped by aspirating the media and washing the cells three times with ice-cold phosphate buffered saline (PBS). The cells were solubilized in 500 μl 0.05 N NaOH for 60 min and the cell-associated radioactivity determined using a liquid scintillation counter (Wallac 1408 Beta Counter). Total cellular protein was determined by the modified Bradford method (28).

To determine the role of phosphatidylinositide 3-kinase (PI3-K) in hormone-dependent glucose uptake, the podocytes were pretreated with 100 nmol/L wortmannin for 5 min prior to stimulation with insulin or AngII. One micro mole per liter chelerythrine (Chel), a specific inhibitor of protein kinase C (PKC), and 1 μmol/L losartan (Los), a specific AngII AT1 receptor antagonist, were added to RM 15 min before the addition of AngII. Phorbol myristate acetate (PMA, 1 μmol/L), a specific PKC activator, was added at the same time as AngII. The reagents were purchased from Sigma-Aldrich, Poland.

### Western blot

Cultured podocytes were harvested by trypsinization and lysed on ice in a pH 8.0 buffer containing 1% Nonidet P-40, 20 mmol/L Tris, 140 mmol/L NaCl, 2 mmol/L EDTA, 10% glycerol, 1 mmol/L sodium orthovanadate, and protease inhibitor cocktail (Complete Mini, Roche Applied Science). For Western blot analysis, 30 μg of total protein was subjected to 8% sodium dodecyl sulfate polyacrylamide gel electrophoresis (SDS–PAGE). Proteins were then transferred to polyvinylidene difluoride (PVDF) Immobilion membranes (Millipore, Bedford, MA, USA) by a semidry blotting system (Hoefer Inc, MA, USA) as described previously (29). Primary antibodies reactive with the insulin receptor β subunit (1:400, rabbit polyclonal, Santa Cruz Biotechnology Inc., CA, USA) and to α-smooth muscle actin (1:2000, mouse monoclonal, Sigma-Aldrich, Poland) were used with secondary antibodies conjugated with alkaline phosphatase (goat anti–rabbit IgG and goat anti-mouse IgG, respectively, Santa Cruz Biotechnology Inc.) The complexes were visualized with 5-bromo-chloro-3-indolyl phosphate (BCIP) and nitro blue tetrazolium (NBT; Sigma-Aldrich, Poland) and photographed with a UVP BioImaging GDS-8000 system (UVP Inc., Upland, CA), using LabWorks 4.0 Image Acquisition and Analysis Software. Analysis of optical densities was performed using Quantity One software (Bio-Rad) and normalized to α-actin.

### Flow cytometry

The cells were washed with PBS, trypsinized, resuspended in PBS, and centrifuged two times for 7 min at 400 × g at room temperature. This was followed by a 60 min incubation at 4°C in blocking solution (2% FBS, 2% bovine serum albumin, 0.2% fish gelatine, in PBS). Finally, the cells were resuspended in cold PBS containing 5% FBS. Aliquots of 3 × 10^5^ cells/tube were incubated for 30 min at 4°C with monoclonal mouse antibodies reactive with the insulin receptor β subunit (1:300, BD Biosciences), with GLUT1 (1:100, Invitrogen), with rabbit polyclonal antibody reactive with GLUT2 (H-67), and GLUT4 (H-61), both at 1:100, (Santa Cruz Biotechnology Inc). The antigen-bound antibodies were visualized by a 30 min incubation at 4°C with Alexa Fluor488-conjugated donkey anti-rabbit and Atto550-conjugated donkey anti-mouse IgG, both 1:100 (Sigma-Aldrich, Poland). Stained cells were washed with 1 ml PBS, resuspended in 300 μl of PBS containing 5% FBS, and analyzed by flow cytometry (FACScan Flow Cytometer, BD, USA). Background fluorescence, assessed with IgG isotype controls, was subtracted from the corresponding samples during analysis.

### Immunofluorescence

Immunofluorescence studies were performed as described previously (30). Briefly, coverslip-attached podocytes were fixed with 2% paraformaldehyde (8 min), and incubated for 60 min in blocking solution as described above. The method was similar to the flow cytometry protocol, except the permeabilization step was omitted in order to visualize surface-bound antibodies. A 60 min incubation with primary mouse antibody reactive with the insulin receptor beta subunit (IRβ subunit (mouse monoclonal, 1:300, BD, USA) was followed by a subsequent 45 min incubation with Atto550-conjugated secondary antibody (1:200, Sigma-Aldrich, Poland). Non-specific staining was controlled by replacing primary antibody with blocking solution alone. The coverslips were mounted on microscope slides using 15% Mowiol solution (Sigma-Aldrich, Poland) and examined by fluorescence microscopy (Olympus IX51), using CellSens v.1.3 imaging software (Olympus). Camera settings were identical for all samples to ensure comparable results.

### Statistical analysis

If not stated otherwise, the data are presented as means ± SEM of 4 to 7 independent experiments completed in triplicate. Results were analyzed using Student's *t*-test, Wilcoxon test, or by one-way analysis of variance (ANOVA). Calculations were completed with Sigma Stat software (version 3.0. for Windows; SPSS Inc., Chicago, IL, USA).

## Results

### Effect of AngII and insulin on the initial velocity of glucose uptake in AT1R+ and AT1R–cells

Glucose uptake by AT1R+ and AT1R- cells in response to insulin and AngII was assessed. Podocytes were incubated for 5 days in NG media and exposed to 1 μmol/L AngII for 30 min or to 300 nmol/L insulin for 20 min, as indicated in Materials and Methods. Initial velocity of glucose uptake was measured with 0.5 mmol/L glucose and 1 μCi ^3^H-DG. Basal (non-stimulated) glucose transport was identical in both cell lines. Both AT1R+ and AT1R- cells significantly increased their glucose uptake in response to insulin but only in AT1R+ podocytes was glucose uptake markedly enhanced by AngII (Table 1). All subsequent experiments were performed with AT1R+ podocytes.

**Table 1:** Initial rate of glucose uptake [nmol/mg/15min].

	**AT1R+**	**AT1R-**
Basal	107 ± 5	103 ± 6
+ Ins	159 ± 7^#^	145 ± 7^#^
+ AngII	144 ± 4^*^^#^	104 ± 6
+ Ins+ AngII	118 ± 8	106 ± 7

*Exposure of podocytes overexpressing AT1 receptor (AT1R+) to insulin or to AngII increased the rate of glucose uptake. Control cells transfected with an empty vector (AT1R-) responded to insulin but not to AngII. Podocytes were cultured for 5 days in NG media and incubated with 0.5 mmol/L glucose and 300 nmol/L insulin (15 min) or with 1 μmol/L AngII (30 min). Results represent the mean ± SEM of four to five experiments in triplicate*.

* *P < 0.05 vs. AT1R-*,

# *P < 0.01 vs. Control*.

### Time dependent effect of AngII on basal and insulin-dependent steady-state glucose uptake

**Figure 1:**
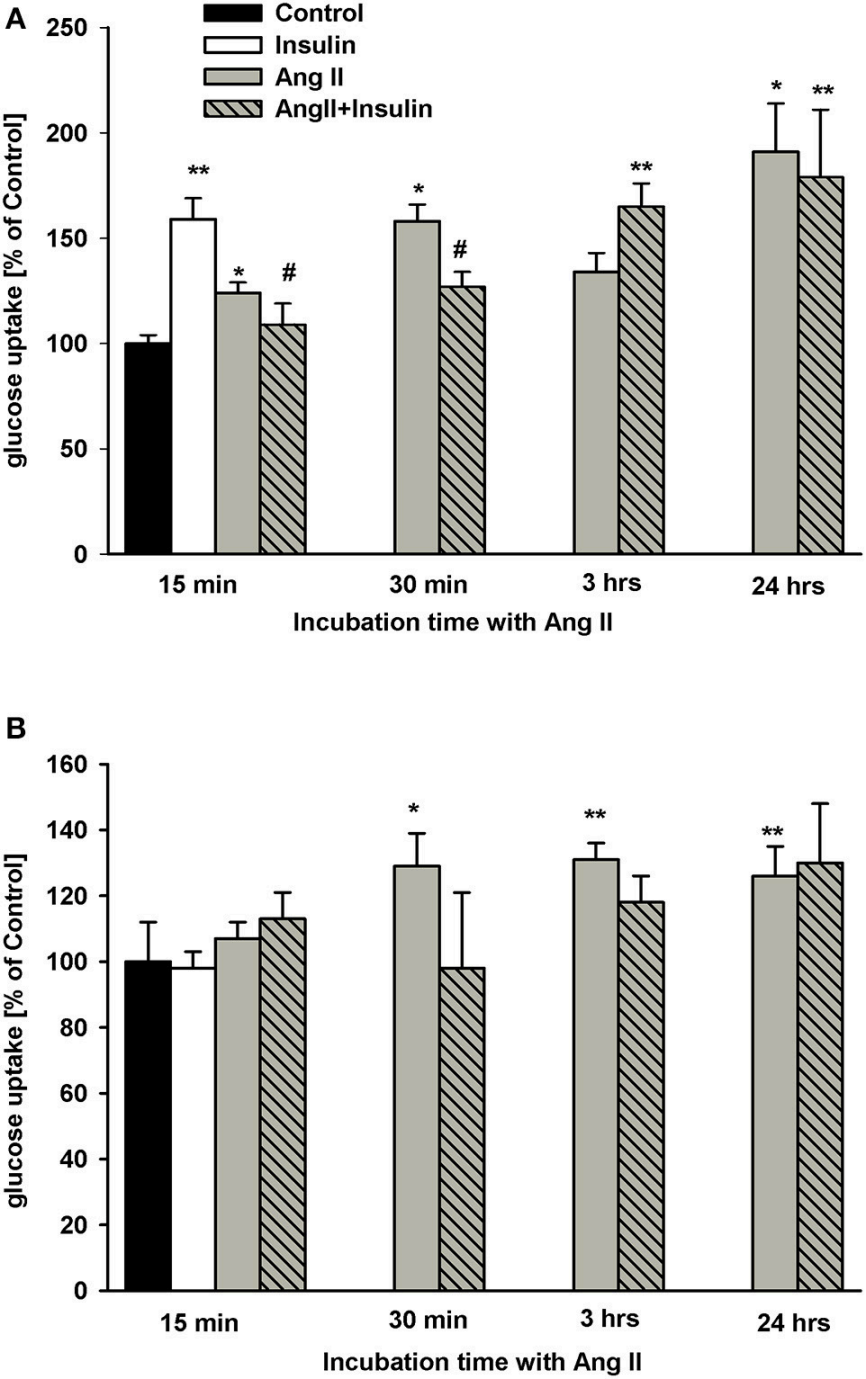
Effect of incubation time with AngII on glucose uptake by podocytes. Podocytes cultured for 5 days in NG **(A)** or in HG **(B)** media were treated with 1 μmol/L AngII for indicated time periods. Three hundred nano mole per liter insulin was added 5 min before ^3^H-2DG and incubations were conducted in NG or HG medium for an additional 15 min as indicated in Materials and Methods. Results are shown as means ± SEM from seven independent experiments. ^*^P < 0.05, ^**^P < 0.01 vs. Control, ^#^*P* < 0.05 vs. insulin.

As shown in Figure 1A, the effects of insulin and AngII on steady-state glucose uptake by NG cells varied based upon podocyte hormone exposure time. After 20 min of incubation, insulin alone increased glucose uptake by 60%. Similarly, AngII alone, incubated with ^3^H-2DG for 15 min, significantly elevated glucose transport. Yet, in the presence of AngII, the effect of insulin was inhibited and glucose uptake was reduced to basal level. For longer exposures of podocytes to AngII, glucose transport was enhanced almost doubling basal levels after 24 h. The inhibitory effect of AngII on insulin-dependent glucose uptake was abolished after 3 h of exposure to AngII.

In podocytes cultured in HG for 5 days, insulin had no effect on glucose uptake (Figure 1B). Further, regardless of preincubation time, AngII did not significantly affect the insulin response. Similarly, no effect of AngII alone was observed after 15 min of incubation. However, glucose uptake significantly increased in HG podocytes treated with AngII for 30 min and up to 24 h.

### Kinetic evaluation of glucose uptake in the presence of AngII and insulin

**Figure 2:**
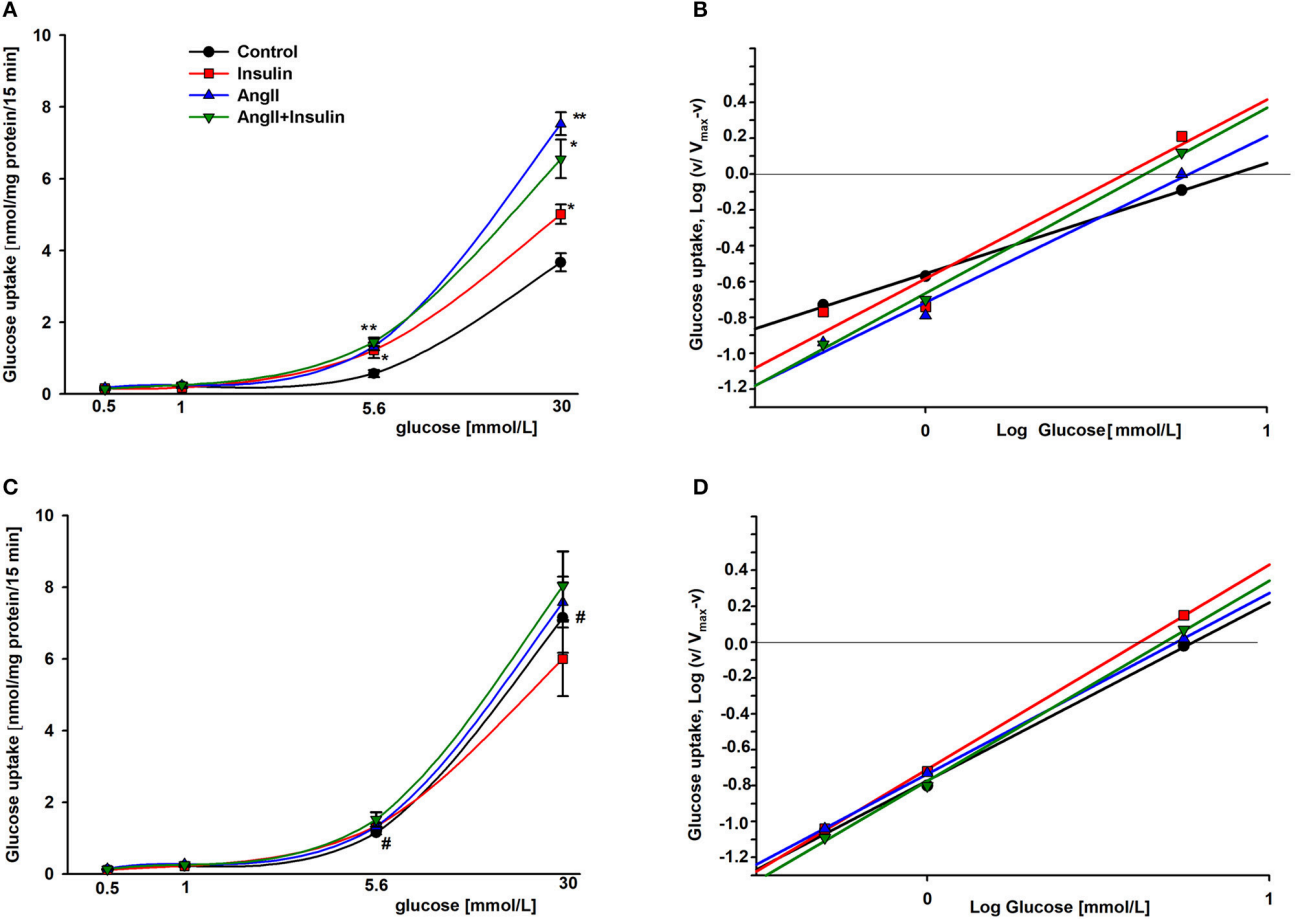
Kinetic evaluation of the effects of AngII on glucose uptake into podocytes incubated for 5 days in NG **(A,B)** or HG **(C,D)**. Panels A and C show the rate of glucose uptake as a function of ambient glucose. Slopes of corresponding Hill plots **(B,D)** are equal to Hill coefficients n_H._ 24 h prior to the glucose uptake measurement, the cells were serum starved in NG or HG media with or without 1 μmol/L AngII. Following a 3-h preincubation in glucose-free media, glucose uptake was measured in reaction media containing RPMI 1640 with or without AngII and supplemented with 0.5, 1.0, 5.6, or 30 mmol/L glucose. 5 min after adding 300 nmol/L insulin, 1 μCi ^3^H-DG was added for the next 15 min, as indicated in Materials and Methods. Data are expressed as the means ± SEM from six independent experiments. ^*^P < 0.05 insulin vs. Control, ^**^P < 0.01 AngII vs. Control, ^#^*P* < 0.05 HG Control vs. NG Control.

To estimate the effect of AngII on glucose uptake dynamics, podocytes were cultured in a physiologic or hyperglycemic milieu. Initial velocity of glucose transport was determined in experimental media containing 1 μCi/ml [^3^H] 2-deoxy-D-glucose and varying concentrations (0.5–30 mmol/L) of glucose, as indicated in Materials and Methods. Kinetic parameters were evaluated using a simplified Hill model. Based on results from our previous kinetic study (31) we made the assumption that glucose transport velocities of 30 mmol/L glucose corresponded to V_max_. In all tested groups, the rate of glucose uptake increased gradually for low ambient glucose concentrations. At a physiological concentration, the rate of ^3^H-2DG accumulation accelerated profoundly for each group (Figure 2). In podocytes cultured for 5 days in HG (Figure 2C), the rate of basal glucose uptake in media containing 5.6–30 mmol/L glucose was markedly higher than that observed with NG cells (Figure 2A). However, with AngII and/or insulin, no significant difference between NG and HG groups was observed.

The S_0.5_ values for total glucose transport (corresponding to Michaelis constant K_m_) were calculated from the Hill equation (Table 2) in which the Hill coefficient was defined by the slope of each line (Figures 2B,D). These values reflect the net activity of the total glucose transport system present in podocytes, comprised of all low-and high affinity transporters. In the cells cultured in NG media, both insulin and AngII clearly decreased S_0.5_ values, demonstrating that the glucose sensitivity of the podocyte transport system was elevated by both hormones. Hill coefficient values for control cells (*n*_H_ = 0.63 ± 0.06) were elevated up to ~1 in the presence of both hormones, indicating that negative cooperativity in the system of basal glucose uptake switched to Michaelis-Menten kinetics in the presence of AngII and insulin. In podocytes cultured in HG, Hill coefficient values were close to 1 in all tested groups, indicating the lack of cooperativity among transporters in the cell membrane. Surprisingly, the S_0.5_ value in non-stimulated control HG cells was lower than in the NG cells (5.78 ± 0.30 vs. 7.68 ± 0.28, P < 0.03), which suggests that the basal affinity for glucose was increased in this group. AngII and insulin had no apparent effect on the affinity of the glucose transport system within the HG group.

**Table 2:** Hill coefficients n_H_ and S_0_._5_ [mmol/L] values for total glucose uptake.

	**Control**		**Insulin**		**AngII**		**AngII** + **Insulin**	
	S_0_._5_	*n*_H_	S_0_._5_	*n*_H_	S_0_._5_	*n*_H_	S_0_._5_	*n*_H_
NG	7.68 ± 0.28	0.63 ± 0.06	3.24 ± 0.10^#^	0.96 ± 0.09	5.63 ± 0.43^*^	1.06 ± 0.05	4.5 ± 0.22	1.07 ± 0.18
HG	5.78 ± 0.30^**^	0.99 ± 0.01	4.97 ± 0.34^$^	1.09 ± 0.03	5.23 ± 0.08	1.01 ± 0.03	5.08 ± 0.26	1.15 ± 0.11

*Podocytes were cultured for 5 days in NG or HG media and pre-incubated for 24 h with 1 μmol/L AngII. 300 nmol/L insulin was added with 1 μCi/ml ^3^H-2DG and glucose uptake was measured for 15 min in media containing 0.5, 1, 5.6, or 30 mmol/L glucose. Results represent the mean ± SEM of six independent experiments in triplicate*.

* *P < 0.05 vs. NG Control*,

$ *P < 0.05 vs. NG Insulin*,

# *P < 0.02 vs. NG Control*,

** *P < 0.03 vs. NG Control*.

### AngII effects on glucose uptake are mediated through AT1 receptors, protein kinase C, and phosphatidylinositide 3-kinase

**Figure 3:**
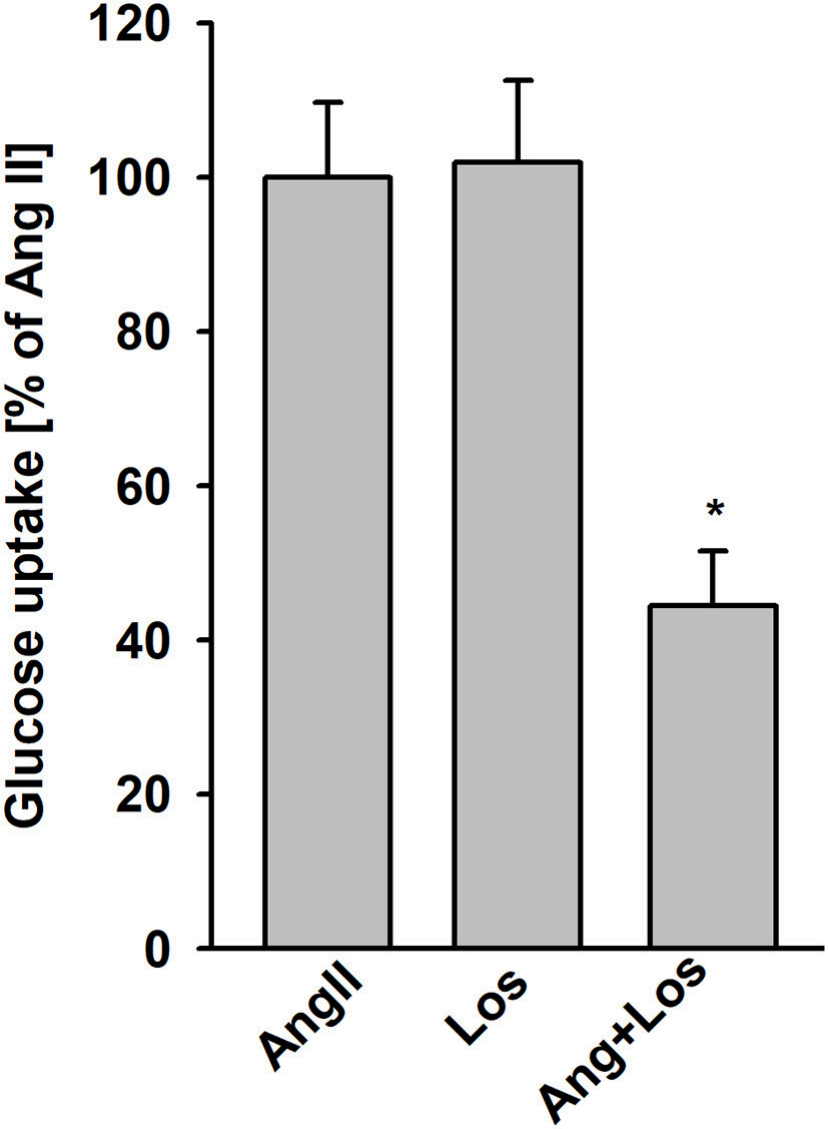
Effect of AngII on glucose uptake mediated by AT1 receptors. Glucose uptake was determined for the steady state conditions in podocytes cultured for 5 days in NG media. One micro mole per liter losartan (Los) was added 5 min before adding AngII. Following a 15 min preincubation with AngII, ^3^H-2DG was added for the next 15 min. Data are expressed as a percentage of the respective control value and are the means ± SEM from four independent experiments. ^*^P < 0.05 vs AngII and Los.

To determine whether AngII stimulation of glucose uptake is mediated by AT1 receptors, podocytes were treated with 1 μmol/L losartan 5 min before addition of AngII. Losartan blocked AngII-stimulated glucose uptake, whereas no effect was observed with losartan alone (Figure 3). These results with those in Table 1 confirm that binding of AngII to the AT1 receptors is required to elicit effects on glucose transport.

**Figure 4:**
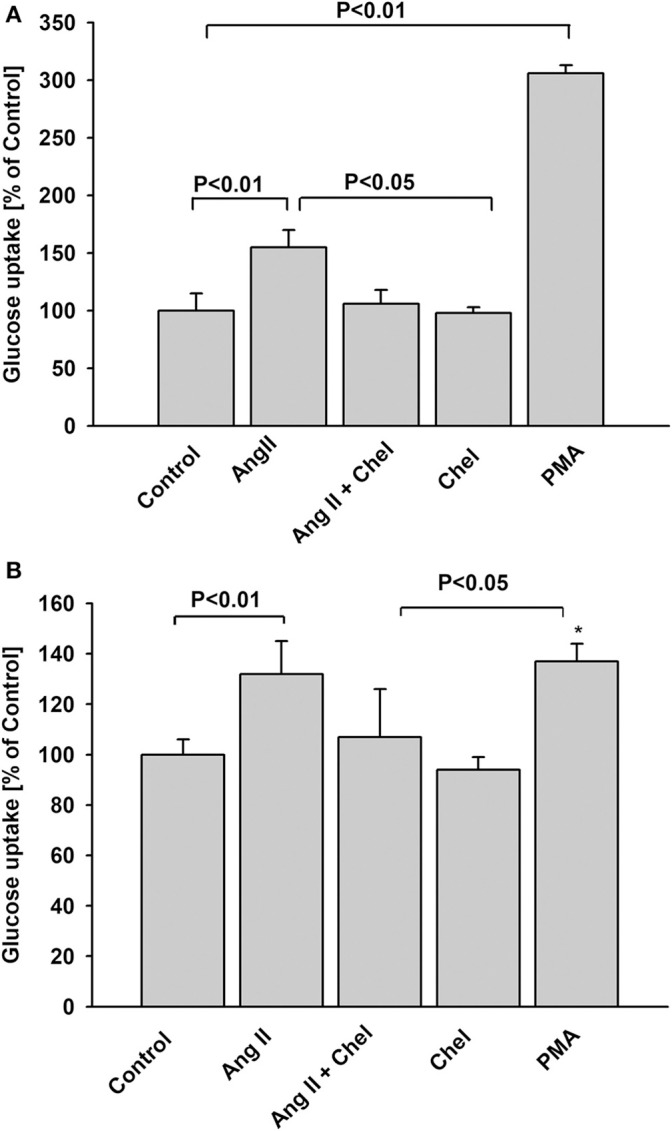
Effect of AngII on glucose uptake mediated by protein kinase C. Glucose uptake was determined for steady state conditions in podocytes cultured for 5 days in NG **(A)** or in HG **(B)** media. One micro mole per liter chelerythrine (Chel) was added 15 min before AngII. One micro mole per liter PMA or 1 μmol/L AngII was added 15 min before ^3^H-2DG and glucose uptake measurement was conducted for the next 15 min. Data are expressed as a percentage of the respective control value and are the means ± SEM from four independent experiments. ^*^P < 0.05 vs Control.

The G-protein-coupled protein kinase C (PKC)-dependent pathway is one of the key mediators of AT1 receptor signaling. Hence, we examined whether PKC plays a role in increased glucose uptake induced by AngII. In both NG and HG groups, pretreatment of podocytes with chelerythrine (Chel), a selective PKC inhibitor completely abolished the effect of AngII (Figures 4A,B). Conversely, incubation of podocytes with the PKC activator, phorbol myristate acetate (PMA), enhanced glucose uptake in NG podocytes. In the HG group the effect of PMA was similar but not as robust as in the NG group. Moreover, there was no significant difference between the effect of AngII or PMA.

**Figure 5:**
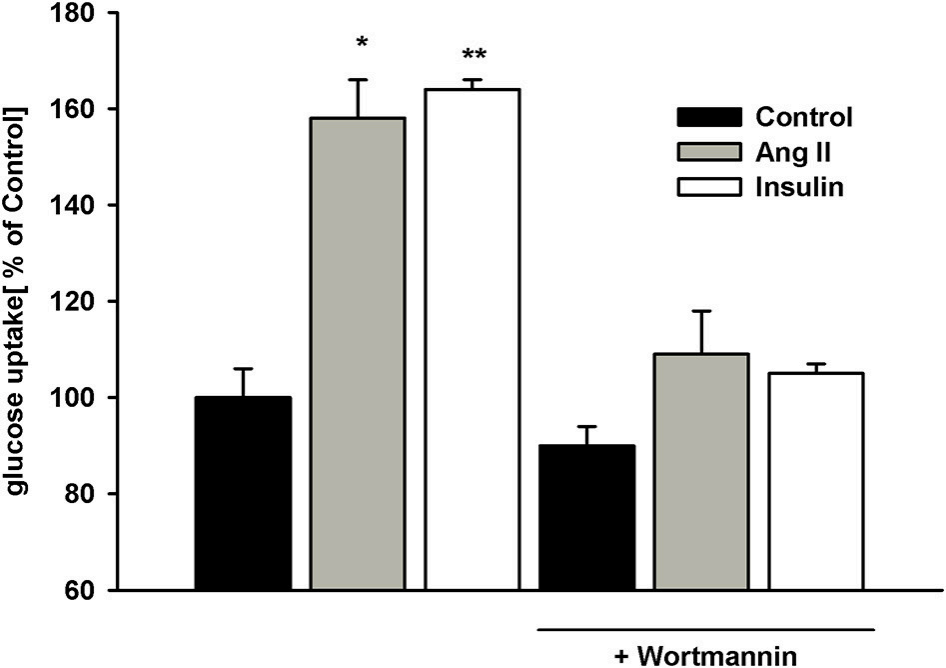
Effect of wortmannin on insulin- and AngII-dependent glucose uptake. Podocytes were incubated for 5 days in NG medium. 100 nmol/L wortmannin was added 5 min prior to insulin or AngII. After a 30-min preincubation with AngII or 5-min preincubation with insulin, glucose uptake measurement was conducted in the presence of ^3^H-2DG in NG medium for 15 min as indicated in Materials and Methods. Data are expressed as a percentage of the control value in the absence of wortmannin and are the means ± SEM from four independent experiments. ^*^P < 0.05 vs. Control and (AngII + wortmannin), ^**^P < 0.01 vs. Control and (insulin +wortmannin).

Phosphatidylinositide 3-kinase (PI3-K) is known to mediate AngII and insulin effects (32, 33). To determine whether PI3-K was involved in glucose uptake modulation herein, podocytes were pretreated with 100 nmol/L wortmannin for 5 min prior to AngII or insulin addition. While wortmannin had no effect on basal (non-stimulated) glucose uptake, stimulatory effects of both AngII and insulin were completely abolished by the PI3-K inhibitor (Figure 5). These results demonstrate PI3-K to be involved in the intracellular signaling that regulates glucose transport in podocytes.

### Effect of AngII on insulin receptor expression

**Figure 6:**
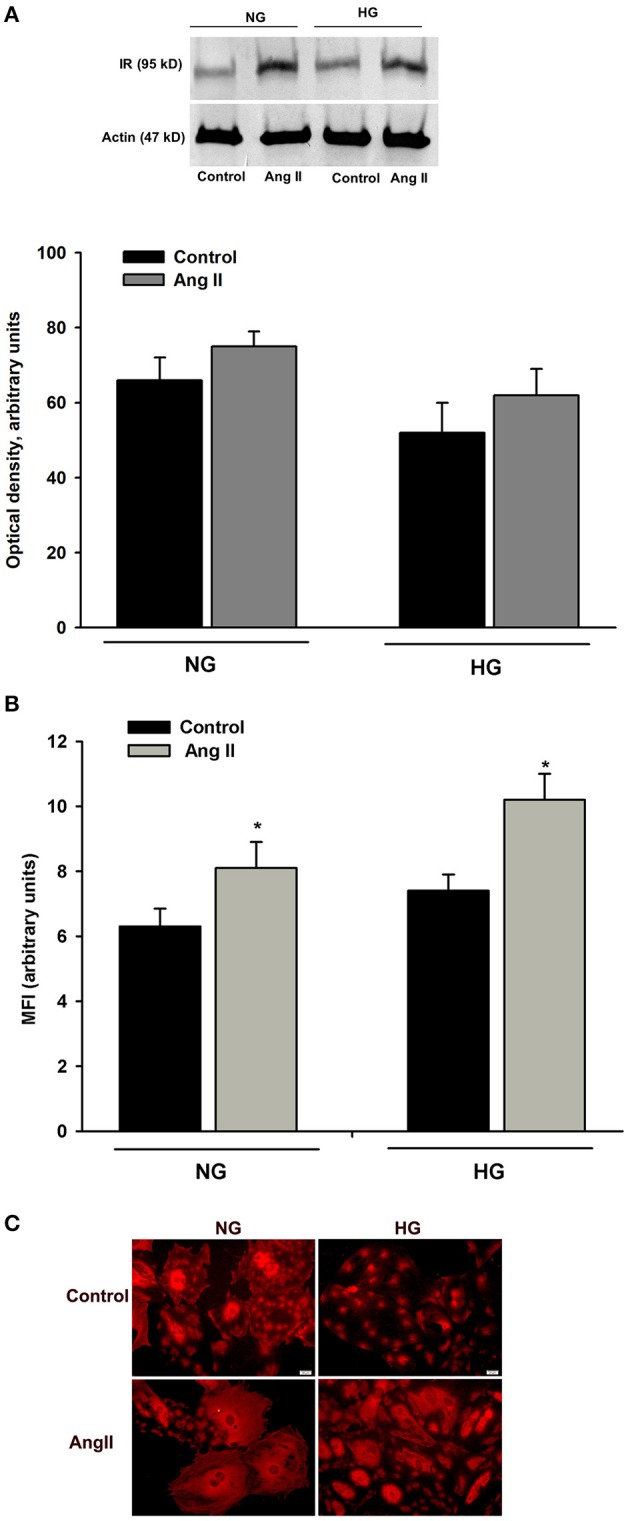
Effect of AngII on IRβ expression in podocytes. Podocytes cultured for 5 days in NG or HG media were incubated for 24 h in corresponding media containing 0.5% FBS, in the presence or absence of 1 μmol/L AngII. **(A)** Western blot analysis was performed with the whole-cell lysates (*n* = 4). **(B)** Flow cytometric analysis demonstrated the expression of IRβ on podocyte surface (*n* = 3). ^*^P < 0.05 vs. Control **(C)**. Immunofluorescent staining of IRβ in non-permeabilized podocytes. Images are representative of three independent experiments.

Glucose transport in insulin-treated cells at 24 h (Figure 1A) was similar to that of cells treated with AngII alone. Thus, it was possible that intracellular glucose accumulation was driven by AngII alone, while insulin signaling was “switched off”. Interactions between AngII and insulin signaling may include changes in insulin receptor numbers. Therefore, we examined whether AngII affected the expression of insulin receptor beta subunit (IRβ) in podocytes cultured for 5 days in NG or in HG media and incubated for 24 h with or without 1μmol/L AngII. As shown in Figure 6A, AngII increased the total amount of IRβ protein in both NG and HG cells. Consistently, flow cytometry results demonstrated that AngII elevated the number of IRβ on the cell surface (Figure 6B). Immunofluorescent microscopic analysis revealed that in non-permeabilized control podocytes, IRβ formed clusters on cell membranes (Figure 6C). After a 24 h incubation with AngII, a majority of the cells expressed IRβ in a diffuse pattern.

### Effect of AngII on GLUT1, GLUT2, and GLUT4 surface expression

**Figure 7:**
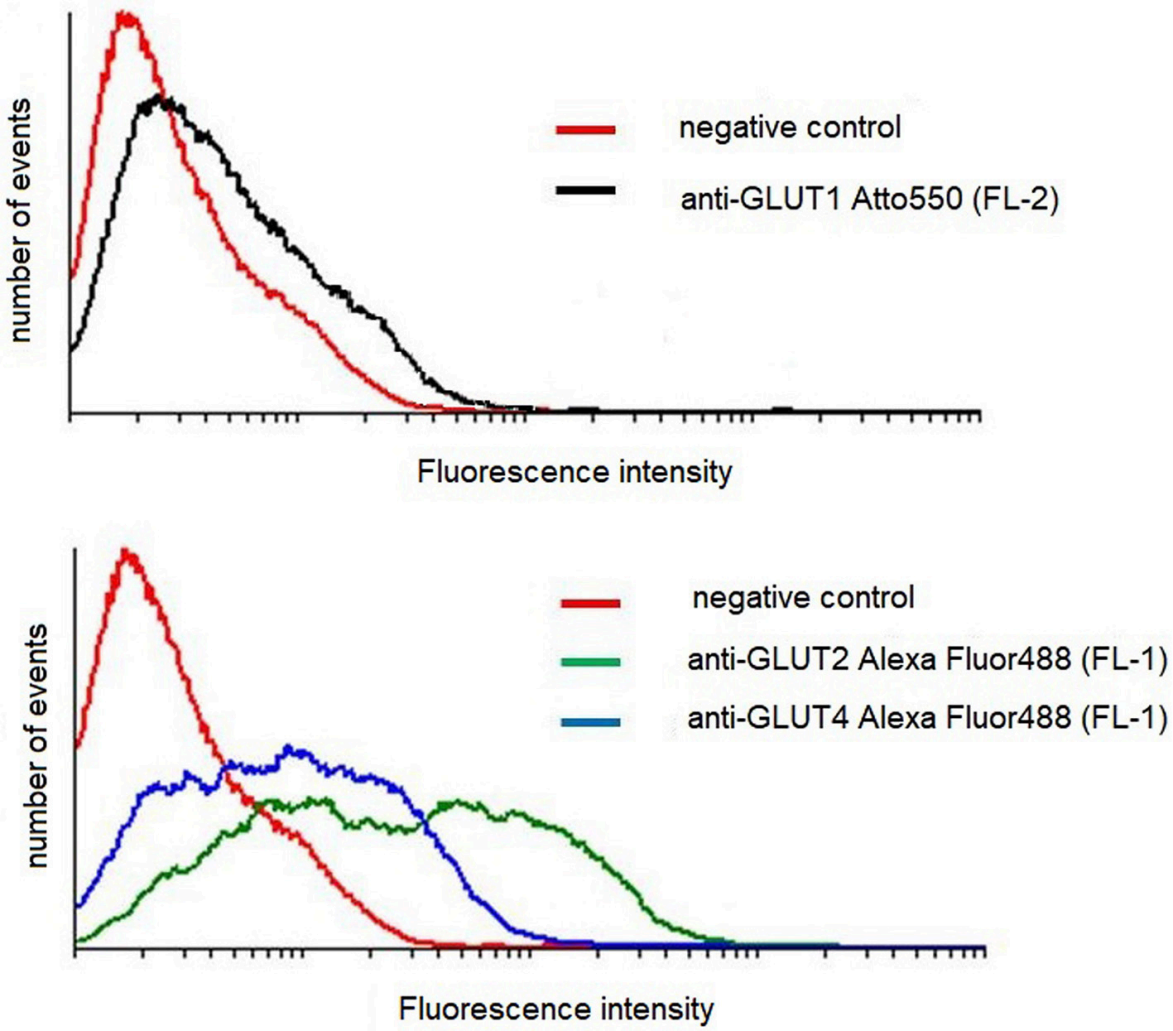
Flow cytometric analysis of GLUT transporters in control mouse podocytes. Cells were stained for surface content of GLUT1, GLUT2, and GLUT4, as described in Materials and Methods. The figure shows results of a representative experiment, which is one of three yielding similar results.

**Figure 8:**
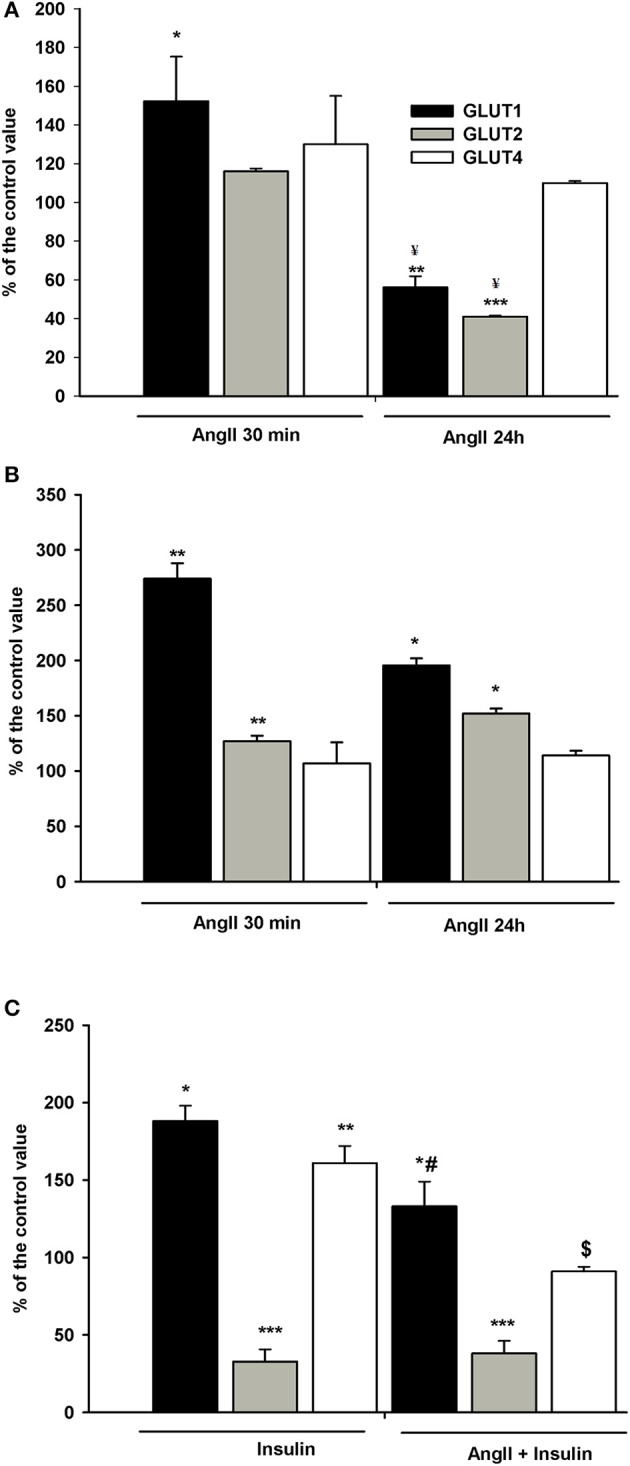
Effects of incubation with AngII on GLUT1, GLUT2, and GLUT4 expression on podocyte surfaces. Podocytes cultured for 5 days in NG **(A)** or HG **(B)** media were incubated for 24 h or for 30 min in corresponding media containing 0.5% FBS, in the presence of 1 μmol/L AngII. **(C)** Effect of AngII on insulin-dependent GLUT1, GLUT2, and GLUT4 expression on podocyte surfaces. Podocytes cultured for 5 days in NG were incubated for 24 h in NG containing 0.5% FBS. 300 nmol/L insulin was added 10 min after adding 1 μmol/L AngII and incubation was continued for the next 20 min. Results of flow cytometric analysis are expressed as the means ± SEM of three independent experiments. ^*^P < 0.05, ^**^P < 0.01, ^***^P < 0.001 vs. Control ^¥^*P* < 0.05 vs. AngII 30 min, ^#^*P* < 0.05 vs. insulin, $ *P* < 0.01 vs. insulin.

Podocytes express a glucose transport system comprised of low- and high affinity glucose carriers (31, 34), hence the effect of AngII on the expression of GLUT proteins in cell membranes was assessed. Interestingly, GLUT2 expression in control, non-stimulated podocytes was apparently higher than that of GLUT1 and GLUT4 (Figure 7). In podocytes from both NG and HG groups, surface expression of GLUT1 increased significantly after a 30 min incubation with AngII (Figures 8A,B). Further, a marked increase in GLUT2 was observed in HG but not in NG cells. There was no significant change in the membrane expression of GLUT4 in both tested groups. After a 24 h incubation with AngII there was a drop in the surface expression of GLUT1 and GLUT2 in the NG podocytes. In contrast, surface expression of both transporters in the HG group remained elevated, as compared to the control group, while expression of GLUT4 remained unchanged in both groups.

Since insulin-stimulated glucose uptake was suppressed in NG cells exposed to AngII for a short period of time (Figure 1A), the effect of AngII on podocyte surface GLUT expression in the cells incubated with insulin was assessed. As shown in Figure 8C, insulin alone elevated expression of GLUT1 and GLUT4 in the cell membrane, while expression of GLUT2 was strongly suppressed. Ten min of AngII pre-incubation prevented the effects of insulin on GLUT1 and GLUT4, while GLUT2 remained unchanged and was similar to the effects of insulin alone.

## Discussion

Previous studies have demonstrated that in some tissues AngII directly modulates the uptake of glucose. However, the observed effects are not consistent and appear to depend on cell type. In cardiomyocytes and adipocytes, AngII had no effect on basal, insulin-independent glucose transport (10, 12). However, AngII was shown to upregulate basal glucose uptake in astroglia, mesangial, and primary vascular smooth muscle cells (4, 5, 35), although one report demonstrated a suppressive effect on a vascular smooth muscle cell line, A10 (9). Further, numerous studies have shown that AngII interferes with insulin-dependent glucose transport activity (10, 36, 37), and as well can produce an insulin-sensitizing effect *in vitro* and *in vivo* (12, 38). In this study, AngII regulation of basal and insulin-dependent glucose transport was examined in podocytes. Since diabetic kidney disease is associated with activation of the intrarenal RAS (3), the impact of glucose concentration on AngII induced glucose uptake was examined.

Results show that brief as well as prolonged (24 h) exposure to AngII significantly enhanced basal podocytes glucose transport (Figure 1). The effect did not depend on glucose concentration, as it was similar in NG and in HG groups. However, it should be noted that in podocytes cultured for 5 days in HG, baseline glucose transport was significantly elevated (223 ± 9 vs. 127 ± 12 nmol/mg protein, *P* < 0.001). After 30 min of incubation with AngII, intracellular glucose accumulation was markedly higher in HG than in NG cultured cells (287 ± 13 vs. 200 ± 12 nmol/mg protein, *P* = 0.003). These results suggest that during hyperglycemia, AngII may potentiate already increased glucose uptake by podocytes. A local podocytes angiotensin system is known to be upregulated by high glucose (21) with multiple pathways proposed for AngII-mediated podocyte impairment during hyperglycemia (21, 39). The results herein suggest that enhancement of gluco-toxicity could be another mechanism by which AngII contributes to diabetic podocytopathy. On the other hand, by enhancing glucose uptake, AngII may support the high metabolic energy demands of podocytes physiologically (40).

Podocytes cultured in HG media develop insulin resistance that manifests as an abrogation of insulin-dependent glucose uptake (Figure 1B), which is consistent with previously reported observations (41). Neither short-term, nor long-term preincubation with AngII affected the response of HG cultured podocytes to insulin. Conversely, insulin-stimulated glucose uptake in NG cultured podocytes was regulated by AngII in a time-dependent manner. A short exposure to AngII (up to 10 min of preincubation) abolished insulin-stimulated glucose uptake (Figure 1A), while with longer preincubation exposures (3 to 24 h), glucose accumulation in insulin-treated cells increased to levels similar to that induced by AngII alone. It is possible that after prolonged exposure to AngII, inhibition of insulin signaling was attenuated and increased glucose uptake was resultant of the actions of both angiotensin and insulin. AngII activation of PKC may be the means by which the insulin-dependent response is modulated. Herein, inhibition of PKC by chelerythrine abolished the effect of AngII on glucose uptake, while PMA, a potent PKC activator, had the opposite effect (Figures 4A,B). These findings clearly demonstrate that in podocytes, PKC is involved in regulation by AngII of glucose uptake. In some animal and human models, PKC activation has been implicated in the development of insulin resistance (42). However, sensitization to insulin after long-term stimulated PKC activity has also been reported (43, 44).

However, with prolonged co-incubation with both hormones, glucose accumulation could be due to AngII alone, while the insulin signaling system was blunted. AngII desensitization of insulin signaling has been well established with multiple mechanisms characterized for angiotensin-induced insulin resistance (32, 45–47). Admittedly, the flow cytometry results demonstrate that in podocytes incubated for 24 h with AngII, the number of surface IRβ receptor subunits was increased (Figure 6B), which may contribute to the restoration of formerly suppressed insulin sensitivity. However, although IRβ receptor number is important, dysregulation of downstream insulin receptor signaling is considered to be a major cause of insulin resistance (48).

For non-stimulated NG podocytes, the Hill coefficient value was below 1 (*n*_H_ = 0.63 ± 0.06, Figure 2, Table 2), which indicates negative cooperativity in glucose binding among the transporters. This may be one of the mechanisms protecting cells from excessive glucose entry. A substantial drop in S_0.5_ values in NG podocytes induced by AngII and insulin (Table 2) indicates that the substrate sensitivity of the glucose uptake system was elevated. In HG cells, the effect of both hormones was not apparent. In our previous study, the estimated V_max_ value for basal glucose transport in rat podocytes was achieved at 10 mmol/L ambient glucose concentration (31). Therefore, in the present study we assumed that at 30 mmol/L glucose for all experimental groups, the V_max_ was already achieved. Given this assumption, calculated V_max_ values for NG podocytes showed that AngII and/or insulin significantly increased the rate of glucose uptake (Figure 2A). This observation, along with reduced S_0.5_ values, demonstrates increased affinity for glucose and is consistent with a hormone-dependent increase in glucose uptake as shown in Figure 1A. In contrast, the maximal velocity of glucose uptake was not affected by the hormones in HG podocytes. Yet, the V_max_ for glucose uptake in control HG cells was almost twice as high as that in control NG cells (7.15 ± 0.98 vs. 3.67 ± 0.25 nmol/mg protein/15 min, *P* < 0.05). Similar high-glucose effects on V_max_ were observed in retinal epithelial and vascular cells (49).

The observed increase in glucose uptake must be due to one of the following; an increase in the total expression of transporters, recruitment of new transporters into the plasma membrane, or activation of the transporters already residing in the plasma membrane. Intracellular glucose accumulation in podocytes is due to the activity of different transporter types (31, 34). GLUT1 is the predominant basal glucose carrier, which along with GLUT4 is involved in insulin-mediated glucose transport (34, 50). Since plasma membrane localization of transporters is essential for glucose uptake, the expression of transporters on the surface podocytes was examined. In both NG and HG podocytes, a short exposure to AngII significantly increased the membrane expression of GLUT1 (Figures 8A,B), which is consistent with increased glucose uptake. However, in contrast to previously published results (51), for NG podocytes incubated with AngII for 24 h, membrane expression of GLUT1 decreased to almost 50% of control values, while intracellular glucose accumulation remained elevated (Figure 1A). It is likely that other mechanisms, such as AngII direct or indirect activation of remaining membrane-bound GLUT1, are possible. The existence of inactive GLUT1 transporters in plasma membranes has been reported previously (52, 53). Other studies have suggested that GLUT1 forms higher-order multimeric complexes on plasma membranes that have enhanced transporter activity (54, 55). GLUT1 has also been identified as a PKC substrate (56) and recent endothelial cell studies demonstrate that AngII via activation of PKC, phosphorylates GLUT1 on S226 with an associated increase in glucose uptake (57). It should also be noted that a 24 h incubation with AngII reduced membrane expression of the low-affinity transporter GLUT2 (Figure 8A). Similar to other polarized epithelial cells, GLUT2 in podocytes enables the basolateral flux of accumulated glucose. Hence, reduction of membrane-bound GLUT2 could contribute to cellular retention of glucose. In contrast, with increased glucose uptake by HG cells, AngII elevated the surface expression of GLUT2 (Figure 8B), which may be a mechanism that protects cells from excessive glucose accumulation.

In NG cells, AngII suppression of insulin-dependent glucose uptake was accompanied by depletion of both GLUT1 and GLUT4 on podocyte surfaces (Figure 8C). This is consistent with previous reports that showed AngII-induced internalization of cell membrane GLUT1 (9) as well as inhibition by AngII of GLUT4 translocation from the cytosol to the cell membrane (36, 58), which may be involved in insulin resistance. Impairment by insulin on GLUT trafficking has been associated with inhibition by AngII of insulin-mediated PI3-K pathway activation (59). AngII signaling pathways also activate PI3-K (60). In this study, not only insulin-dependent but also AngII-dependent glucose uptake in podocytes was mediated by PI3-K (Figure 5). However, competitive cross-talk between AngII and insulin is complex and may involve other signal transduction proteins involved in glucose uptake modulation (32).

In summary, these data clearly demonstrate that AngII, via the AT1 receptor and the PKC and PI3-K signaling pathways, enhances basal glucose uptake in podocytes cultured in normal- and in high-glucose media. Moreover, despite upregulation by AngII of the IRβ receptor expression on cell surfaces, AngII inhibits insulin-dependent glucose transport of cells cultured in NG conditions. The effects of AngII on basal and on insulin-dependent glucose uptake by podocytes was mediated by modulation of a cell membrane-bound system of glucose transporters. With high ambient glucose, podocyte glucose transport system becomes insensitive to insulin and AngII does not affect its insulin sensitivity.

Based on the results herein, the physiological role of AngII is to provide additional energy for podocytes to meet the high metabolic demands experienced by these cells. However, AngII stimulated intracellular glucose accumulation in a high-glucose environment may be a mechanism by which glucose and AngII impair podocytes. Suppression of insulin-dependent glucose transport suggests that activation of RAS in podocytes may contribute to the development of insulin resistance.

In this study we have not examined AngII induced changes in the expression of glucose transporters due to translational and/or post-translational modifications, regulation of GLUT trafficking, or both. Nor have we evaluated the interactions between AngII and insulin that modulate glucose transport in podocytes. Future investigations will explore these issues.

## Author contributions

BL contributed to the concept and design of the study, the analysis and interpretation of data, as well as the preparation of the manuscript draft. AM, EL, and AD generated data. BL and AD completed statistical analysis. AR critically revised the manuscript.

### Conflict of interest statement

The authors declare that the research was conducted in the absence of any commercial or financial relationships that could be construed as a potential conflict of interest.
